# Microarray Profiling of Mononuclear Peripheral Blood Cells Identifies Novel Candidate Genes Related to Chemoradiation Response in Rectal Cancer

**DOI:** 10.1371/journal.pone.0074034

**Published:** 2013-09-05

**Authors:** Pablo Palma, Marta Cuadros, Raquel Conde-Muíño, Carmen Olmedo, Carlos Cano, Inmaculada Segura-Jiménez, Armando Blanco, Pablo Bueno, J. Antonio Ferrón, Pedro Medina

**Affiliations:** 1 Division of Colon and Rectal Surgery, HUVN, Granada, Spain; 2 Departments of Biochemistry, Molecular Biology and Immunology, University of Granada, Granada, Spain; 3 Department of Surgical Research, HUVN, Granada, Spain; 4 Department of Computer Science and Artificial Intelligence, University of Granada, Granada, Spain; ENEA, Italy

## Abstract

Preoperative chemoradiation significantly improves oncological outcome in locally advanced rectal cancer. However there is no effective method of predicting tumor response to chemoradiation in these patients. Peripheral blood mononuclear cells have emerged recently as pathology markers of cancer and other diseases, making possible their use as therapy predictors. Furthermore, the importance of the immune response in radiosensivity of solid organs led us to hypothesized that microarray gene expression profiling of peripheral blood mononuclear cells could identify patients with response to chemoradiation in rectal cancer. Thirty five 35 patients with locally advanced rectal cancer were recruited initially to perform the study. Peripheral blood samples were obtained before neaodjuvant treatment. RNA was extracted and purified to obtain cDNA and cRNA for hybridization of microarrays included in Human WG CodeLink bioarrays. Quantitative real time PCR was used to validate microarray experiment data. Results were correlated with pathological response, according to Mandard´s criteria and final UICC Stage (patients with tumor regression grade 1–2 and downstaging being defined as responders and patients with grade 3–5 and no downstaging as non-responders). Twenty seven out of 35 patients were finally included in the study. We performed a multiple t-test using Significance Analysis of Microarrays, to find those genes differing significantly in expression, between responders (n = 11) and non-responders (n = 16) to CRT. The differently expressed genes were: BC 035656.1, CIR, PRDM2, CAPG, FALZ, HLA-DPB2, NUPL2, and ZFP36. The measurement of FALZ (p = 0.029) gene expression level determined by qRT-PCR, showed statistically significant differences between the two groups. Gene expression profiling reveals novel genes in peripheral blood samples of mononuclear cells that could predict responders and non-responders to chemoradiation in patients with locally advanced rectal cancer. Moreover, our investigation added further evidence to the importance of mononuclear cells’ mediated response in the neoadjuvant treatment of rectal cancer.

## Introduction

Preoperative chemoradiotherapy is the recommended standard therapy for patients with locally advanced rectal cancer (LARC). However, recently studies suggested that preoperative chemoradiotherapy (CRT), as compared with postoperative chemoradiotherapy, improved local control and were associated with reduced toxicity [1]–[6]. After neoadjuvant CRT the ability to achieve pathologic downstaging, or a complete pathologic response, is correlated with improved survival, decreased local recurrence, and a higher rate of sphincter-preserving surgeries [7]–[9].

Approximately 40–60% of LARC patients treated with neoadjuvant CRT achieve some degree of pathologic response. However, there is no effective method of predicting which patients will respond to neoadjuvant CRT. Prospective identification of patients who have a higher likelihood of responding to preoperative CRT could be important in deceasing treatment morbidity and improving survival and local control in LARC. In addition, patients who are unlikely to respond could be offered alternative approaches to therapy.

Peripheral blood mononuclear cells (BCs) comprise the circulating mononuclear cells, including monocytes, T-cells, B-cells, and natural killer cells, and have emerged in recent years as surrogate markers of several diseases including inflammatory (e.g. rheumatoid arthritis, and chronic pancreatitis) and malignant diseases like renal cell carcinoma [10]–[12]. However, in contrast to tissue markers, their role in prediction and prognostic assessment of solid tumors remains limited to recent investigations using gene chips which focus on breast, esophageal, pancreatic and colorectal cancers [13]–[16].

In the present study, we test if the gene expression profile of BCs could identify response to CRT and, therefore, be a predictor marker in the multidisciplinary treatment of patients with LARC.

## Materials and Methods

### Patients and Tumour Characteristics

The group of study initially consisted of 35 locally advanced rectal cancer (LARC) patients from the Division of Colon & Rectal Surgery, HUVN, Granada, Spain, with additional 8 patients of the validation group. To qualify for this study, rectal carcinomas had to be on the stage II or stage III according the criteria of the International Union Against Cancer’s (UICC), without systemic metastases in the positron emission tomography scan and no known second neoplasm. The diagnosis of rectal cancer was confirmed by the histopathological analysis of endoscopic biopsies. The study was approved by the Hospital Universitario Virgen de las Nieves - Granada ethics committee. Written informed consent was obtained from all patients before the study. After the initial staging, all patients qualifying for this study received neoadjuvant radiotherapy (28 fractions of 1.8 Gy, 5 fractions/week) with concomitant chemotherapy (capecitabine, 825 mg/m^2^, twice daily alone or in combination with oxaliplatine 50 mg/m^2^ once weekly). Standardised surgery, including total mesorectal excision, was performed 8 weeks after the standardised CRT protocol described above.

Standard pathologic tumor staging of the resected specimen was performed according to UICC guidelines and the tumour regression grade (TRG). Mandard classifications were assigned by at least one specialized gastrointestinal pathologist. For this study, patients with TRG 1 and 2 and downstaging were considered as responders whereas patients classified with regression grades 3 to 5 and no downstaging were treated as non-responders. Downstaging was defined as reduction of pathologic staging (ypStage) in relation to pretreatment stage (cStage) (i.e., cII to ypI, cIII to ypII or ypI).

### Isolation of RNA from BCs

Total RNAs were extracted from 12 mL of peripheral blood mononuclear cells (BCs) samples before treatment from each study participant using PAXgene Blood RNA System (PreAnalytix, Becton Dickinson, San Diego, CA, USA) according to manufacturer’s instructions. Quantity and integrity of RNAs were checked by spectrophotometry in a NanoDrop™ (ND-1000, DE, USA) and in an Experion™ automated electrophoresis system (Bio-Rad, Richmond, VA, USA), respectively.

### Gene Expression Analysis

In brief, following reverse transcription cRNAs were labeled with Cy5 Streptavidine (Amersham Biosciences, Sweden). Hybridization of whole genome human genes included in CodeLink bioarrays (Applied Microarrays, Tempe, AZ, USA) was performed overnight at 37°C in a shaker incubator (Innova 4080, New Brunswick®, NJ, U.S.A.). The hybridization reactions were done in duplicate. Microrrays were read with a GenePix 4000B laser scanner (Axon Instruments, CA, USA), quantified and normalized using CodeLink Software 5.0 (Applied Microarrays, Tempe, AZ, USA). Microarray data were normalized using different methods: average normalization and cyclicLoess. The quality of the outcome was assessed by different plots produced by the software package ArrayQualityMetrics implemented in the R language. A supervised method (Significance Analysis of Microarrays -SAM-) [17] was then used to find the more significant (adjusted p<0.05) differentially expressed genes in rectal cancer patients who responded to treatment and those who did not.

Upon concluding this process the raw gene expression values were obtained for each of the samples. This dataset has been made publicly available at the Gene Expression Omnibus GEO database [18] with submission number GSE44172.

### Correlation of Gene Signatures to CRT Response

Samples were grouped into the two categories according to response to treatment. The differential expression of genes was then evaluated for the proposed categories using the software SAM (Significance Analysis of Microarrays, Stanford University, CA, USA) [17]. The cut-off for significance was determined by tuning the parameter delta (Δ), which was chosen based on the False Discovery Rate (FDR). Genes with corrected p-values <0.05 were considered as significantly differentially expressed between the groups.

### Validation of Microarray Data by qRT-PCR

We used quantitative Real Time PCR (qRT-PCR) to confirm some of the results found in the microarray gene expression profile. We optimized a sensitive and specific qRT-PCR assay using MX3005P QPCR System (Stratagene, Agilent Technologies, La Jolla, CA, USA). One microgram of RNA was used for reverse transcription with QPCR-grade AffinityScript® Multiple Temperature Reverse Transcriptase (AffinityScript® QPCR cDNA synthesis kit, Stratagene, Agilent Technologies, La Jolla, CA, USA) using random hexamers. PCR reactions contained 1 µg cDNA, 12.5 µL Brilliant II® qPCR Master Mix (Stratagene, Agilent Technologies, La Jolla, CA, USA), 1.25 µL Solaris® (Dharmacon, Thermo Scientific, Chicago, IL, USA) primer/probe set for each gene. PCR conditions were 15 min at 95°C, 15 s at 95°C and 1 min at 60°C for 40 cycles. We designed specific Taqman® probes and primers (Table 1). Before performing this study GAPDH (RT-CKYD-GAPD, Yakima Yellow® Eclipse Dark Quencher®), RPL13A (RT-CKYD-RPL13A Yakima Yellow® Eclipse Dark Quencher®) and TBP (RT-CKYD-TBP Yakima Yellow® Eclipse Dark Quencher®) genes were selected as a candidate housekeeping gene. Despite the fact that GAPDH is over-expressed in BCs of several malignancies such as cervical and ovarian cancers [19], GAPDH emerged as the most stable gene, with no closely comparable housekeeping gene among the evaluated genes in a series of tumors.

**Table 1:** Specific Taqman® primers and probes used in quantitative RT-PCR assays of over-expressed genes in BCs samples from responder rectal cancer patients before treatment.

Gene Name	Name	Sequence
**CAPG**	Probe	TCAAGTACCAGGAAGGT
	Forward primer	CAATGAGTCTGACCTCTTC
	Reverse primer	GTGAAATGCTGACTCCACACCA
**CIR**	Probe	GTCTTTCTGGAATCAATG
	Forward primer	CAGAGATCAGCCCTTTGGTA
	Reverse primer	GTGGGAACCGAACTTGCATT
**FALZ**	Probe	ATAGTACCTACAGCAGC
	Forward primer	GACGACGATGACTCCGATT
	Reverse primer	TTTTCGCCTACCTGGAGTG
**NUPL2**	Probe	AGCAATAACTTACAGAG
	Forward primer	GGTTTTACAGACATTTCACCAG
	Reverse primer	CGTTGGACAGAATTTAGATAACTC
**PRDM2**	Probe	AACCCTGAGATAGCAGCT
	Forward primer	CTCCTGGTCTGGTACAATG
	Reverse primer	TCGCTCTTCCTCAATCGCA
**ZFP36**	Probe	CCGTGCCATCCGACCAT
	Forward primer	TGCCATCTACGAGAGCCT
	Reverse primer	GGACTCAGTCCCTCCAT

Expression was quantified following the analysis of two different dilutions of cDNAs (1 and 1/10) in triplicate. For each experimental sample, the amount of the each gene and endogenous reference (GAPDH) was determined from the standard curves. These standard curves were composed of five points obtained from five-fold serial dilutions (1, 1/10, 1/50, 1/100, and 1/500) of cDNA from Universal Human Reference RNA (Stratagene, Agilent Technologies, La Jolla, CA, USA). It is composed of total RNA from 10 human cell lines. We considered only experiments in which the linear relationship between Ct (threshold cycle) and the log of the amount of standard curve for each gene and GAPDH were higher than 0.99 (correlation coefficient). The expression values of each gene were then divided by the amount of GAPDH to obtain a normalized value. GAPDH gene was used as an internal control for RNA quality reverse transcription and to correct the variations in the degree of RNA degradation. Statistical significance of differences in transcript levels was assessed using the non-parametric T-test (Mann Whiteney). Data analyses were carried out with the SPSS statistical software, version 15.0 (SPSS Inc., Chicago, IL, USA). To perform quantification of the expression of genes, a standard curve was constructed with at least four different concentrations in triplicate. The GAPDH gene was used as control gene to test the quality of cDNAs. Expression was estimated after the analysis of two different dilutions of the cDNAs, each one of the dilutions analyzed in triplicate. Differences in gene expression between Responder and Non-responder groups were estimated using non-parametric T-test.

## Results

### Patients and Tumor Characteristics

Eight of the 35 initial patients were finally excluded due to the poor quality of the RNA or contradictory results of Mandard´s criteria and histopathological downstaging. Statistical power of more than 0.8 was obtained with these 27 patients.

Clinical data of the final 27 patients are shown in Table 2, further including the 8 patients of the validation group (patient 28 to 35). Note that the postoperative UICC stage represents tumor stage after neoadjuvant treatment (ypT). No statistically significant differences (chi square) were found in terms of CRT, surgery or sex when comparing between the two groups (response vs. non-response) (Table 3). Furthermore, no significant differences (t-student) were found in total leukocyte number and haemoglobin between both groups (responders and non-responders) (Table 4).

**Table 2:** Patients and tumour characteristics.

Pat N	Sex	Age	CRT	cTN	Surg	TRG	Downst	Resp	Leuc	Lymp
1	Male	63	capox	T4N1	LAR	2	Yes		5,2	34,4
2	Male	71	cap	T3N0	LAR	2	Yes	Yes	8,4	17
3	Male	77	cap	T3N1	LAR	4	Yes	Yes	8,4	25,3
4	Male	67	cap	T3N0	LAR	5	No	No	6,8	25,7
5	Female	83	cap	T3N0	APR	2	Yes	No	5,1	32,1
6	Female	63	cap	T3N2	LAR	5	No	Yes	6,7	39,6
7	Male	51	capox	T4N0	APR	2	Yes	No	9,7	22,2
8	Male	53	capox	T3N1	LAR	1	Yes	Yes	7	32,1
9	Male	64	capox	T3N2	HART	2	No	Yes	8,5	24,5
10	Male	69	cap	T3N0	HART	3	Yes	Yes	8,9	27,7
11	Male	69	cap	T3N0	LAR	1	Yes	No	6,9	33,1
12	Male	59	cap	T3N0	APR	4	Yes	Yes	7,9	28,6
13	Male	71	cap	T3N0	LAR	5	Yes	No	7,7	32,3
14	Female	62	cap	T3N1	HART	5	Yes	No	12,2	12,3
15	Female	58	cap	T3N0	LAR	1	Yes	No	7,1	47
16	Male	50	capox	T4N0	APR	4	No	Yes	8,5	12,5
17	Male	36	capox	T4N0	HART	5	No	No	11,6	36,2
18	Male	47	capox	T3N0	LAR	4	No	No	9,8	21,1
19	Male	45	capox	T3N0	APR	5	No	No	10	20,4
20	Male	47	capox	T3N1	HART	1	Yes	No	9	17,3
21	Male	74	cap	T3N0	HART	4	Yes	Yes	6	26,6
22	Female	61	cap	T3N1	LAR	4	No	Yes	2,1	33,1
23	Female	37	capox	T3N2	LAR	5	No	No	5,2	32
24	Male	54	cap	T3N0	LAR	1	Yes	No	7,6	34,8
25	Male	69	capox	T3N2	APR	3	No	Yes	5,7	21,6
26	Female	70	cap	T3N2	LAR	3	Yes	No	8	25,8
27	Male	61	capox	T3N1	LAR	2	Yes	No	8	30,4
28	Female	76	cap	T3N0	LAR	4	Yes	Yes	5,24	16,6
29	Female	64	cap	T3N2	LAR	4	No	No	7,64	12,3
30	Male	63	cap	T3N1	LAR	2	Yes	No	4	18,3
31	Female	56	cap	T2N1	LAR	3	Yes	Yes	5,66	26,5
32	Male	62	cap	T3N1	LAR	4	No	No	4,19	21,7
33	Male	64	cap	T3N2	LAR	3	No	Yes	3,25	17,8
34	Male	56	cap	T4N1	LAR	3	Yes	No	6,21	15,6
35	Male	62	cap	T3N1	LAR	4	No	No	10,44	5,1

CRT: Chemoradiation; Cap: Capecitabine; Capox: Capecitabine and Oxaliplatine; cTN: clinical stage, Surg: surgical technique, LAR: Low anterior resection, APR: Abdmino-perineal resection; HART: Hartmann, TRG: Tumor Regression Grade; Downst: Downstaging; Resp: response, Leuc: leucocytes (×10^3^×ml), Lymp: lymphocytes (%).

**Table 3:** Clinical data comparing both groups (responder and non-responder). Data are presented as number of patients (percentage).

Measurement	Group	Mean values ± SE	
Leukocytes (×1000/µL)	Responders	7.2±1.17	p = 0.902
	Non-responders	7.31±2.54	
Haemoglobin (g/dL)	Responders	14.93±1.96	p = 0.240
	Non-responders	13.76±2.78	
Age (years)	Responders	61.4±9.9	p = 0.863
	Non-responders	60.7±11.3	

**Table 4:** Leukocytes (10^3^/µL), haemoglobin (g/dL) and age values in responder (n = 12) and non-responder (n = 23) rectal cancer patients before treatment.

	No response 23(65.7%)	Response 12 (34.3%)	p
**Drug**			0.261
Capecitabine	17 (73.9%)	6 (50.0%)	
Capecitabine+ oxaliplatine	6 (26.1%)	6 (50.0%)	
**Surgical technique**			1
Anterior resection	19(82.6%)	10 (83.3%)	
Abd-perineal resection	4 (17.4%)	2 (16.7%)	
**Sex**			0.434
Women	8(34.8%)	2 (16.7%)	
Men	15 (65.2%)	10(83.3%)	

Data are presented as mean values ± SD (standard deviation).

### Differential Gene Expression in Peripheral Blood Samples between Treatment Responder and Non-responder Patients with Rectal Tumour

A supervised method (Significance Analysis of Microarrays -SAM-) was used to search for a gene signature showing significant differences between expression profiles for responder and non-responder patient subgroups. We found that 8 genes were differentially expressed (p<0.05) in BCs from responder and non-responder samples. All these genes presented significantly higher expression levels (over-expression) in responder LARC patients. Table 5 describes these 8 genes (BC035656.1, CIR, PRDM2, CAPG, FALZ, HLA-DPB2, NUPL2, and ZFP36). Genes BC035656.1, CIR and PRDM2 showed the highest difference of gene expression values between the two groups (Log-ratio: 1.47, 1.34 and 1.24 respectively).

**Table 5:** Genes over-expressed in peripheral blood mononuclear cells from locally advanced rectal cancer patient responders.

Gene Name	Log-ratio	Description	Code Link ID
**BC035656.1**	1.47	hypothetical protein LOC285835, mRNA (cDNA clone IMAGE:5588650). Discontinued	BC035656.1
**CIR**	1.34	CBF1 interacting corepressor (CIR). transcript variant 1	NM_004882.3
**PRDM2**	1.24	UI-H-BW1-and-f-10-0-UIs1 NCI_CGAP	BF514317.1
**CAPG**	1.24	capping protein (actin filament). gelsolin-like (CAPG)	NM_001747.2
**FALZ**	1.14	fetal Alz-50-reactive clone 1 (FAC1)	U05237.1
**NUPL2**	0.82	xm72b03x1 NCI_CGAP_Kid11	AW237453.1
**ZFP36**	0.79	zinc finger protein 36. C3H type	NM_003407.1
**HLA-DPB**	0.72	NIA Human H1 Embryonic Stem Cell cDNA Library (Long)	CD655061.1

### Quantitative RT-PCR Validation

mRNA expression levels of six of the genes (CIR, PRDM2, CAPG, FALZ, NUPL2, and ZFP36) over-expressed in treatment responder patients were analyzed by qRT-PCR. HLA-DPB2 and BC035656.1 were discarded due to complexity of the HLA-DP region and sequence discontinued respectively.

**Figure 1:**
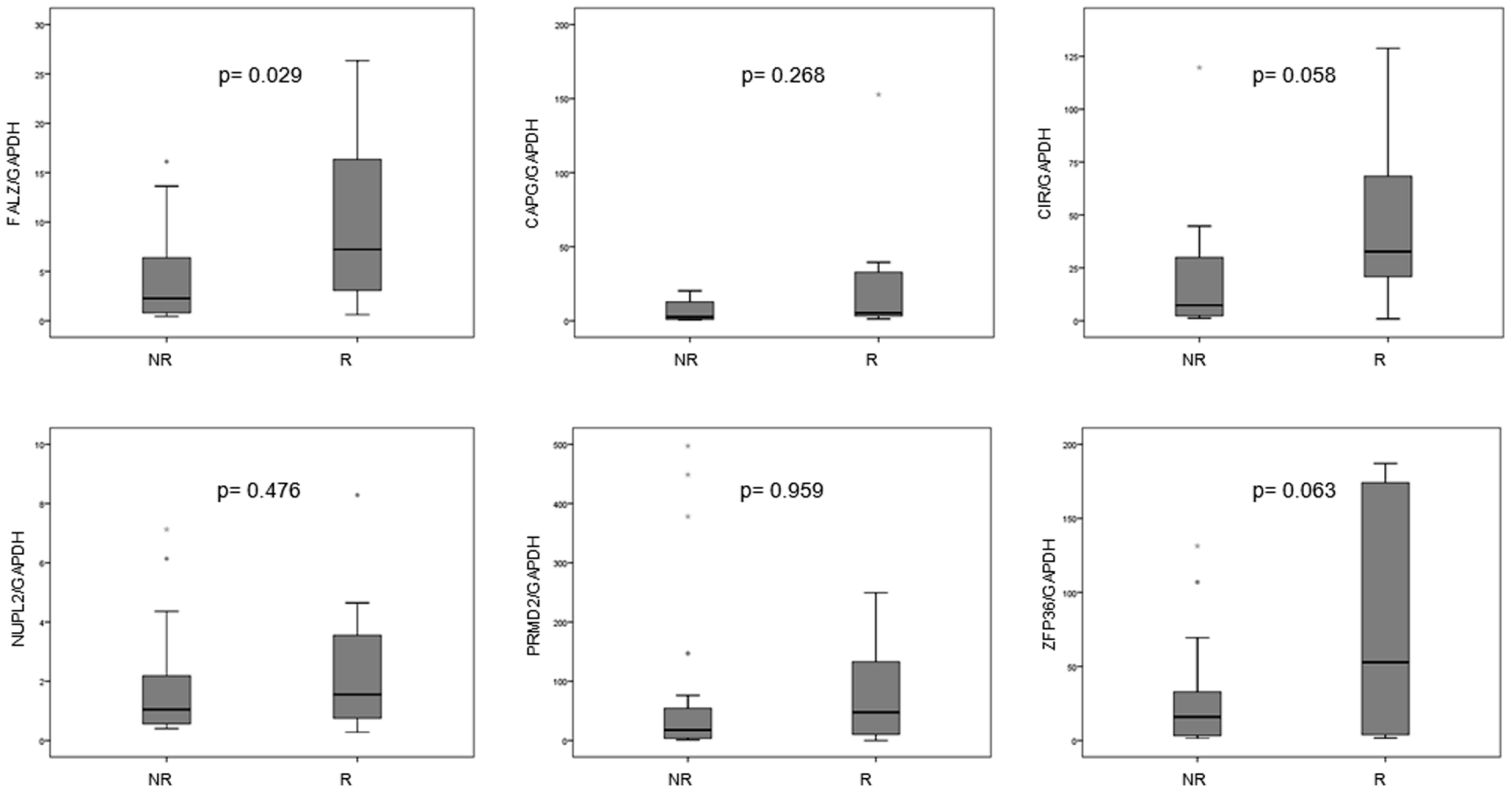
Box plots representing expression values of FALZ, CAPG, CIR, NUPL2, PRDM2, and ZFP36 genes by quantitative real-time RT-PCR in both groups of rectal cancer patients defined by their response to treatment: responder (R), and non-responder (NR). FALZ, CAPG, CIR, NUPL2, PRDM2, and ZFP36 expression levels were successfully obtained from 30, 13, 29, 27, 30, and 23 LARC patients. Boxes represent the quartiles, median is represented by a black line within the box, and circles (0) show atypical values (1.5–3 times the length of the box). Asterisk (*) shows extreme values (more than three times the box). FALZ gene expression showed statistically significant differences between responder and non-responder patients.

The measurement of gene expression levels determined by microarray analysis positively correlated with the qRT-PCR analysis. Both microarray and qRT-PCR results confirmed similar trends of gene expression profiles of selected genes in rectal cancer patients. As occurred in the microarray expression analysis, we found significant differences between the expression of FALZ gene (p = 0.029) in treatment responder and non-responder groups. CAPG (p = 0.268), CIR (p = 0.058), NUPL2 (p = 0.476), PRDM2 (p = 0.959), and ZFP36 (p = 0.063) showed higher expression in responder patients, without ever reaching statistical significance (Figure 1).

### Validation of Predictive Biomarkers

To evaluate the prognostic relevance of FALZ, CIR and ZFP36 genes, we applied qRT-PCR. In order to reach a sufficient power in the analysis, we have added the validation cohort (n = 8) to the overall population studied (n = 27). According to the criteria used (patients with Mandard´s TRG 1 and 2 were considered as responders), only one of them was classified as a responder. For each mRNA (array and qRT-PCR data) a receiver operating characteristic (ROC) curve was generated. Area under curve (AUC) value and 95% confidence interval (CI) were calculated to determine the specificity and sensitivity of response to treatment prediction. ROC curves of FALZ microarrays’ data reflected moderate ability to distinguish between the responder subgroup and Non-responder subgroup, with an AUC of 0.843. At a cut-off point set at 20.72, FALZ yielded a sensitivity of 80.0% and a specificity of 85.7%. ROC curves of FALZ qRT-PCR data had an AUC of 0.681.

## Discussion

Modern oncological treatment decisions increasingly depend on so-called clinical and laboratory predictive and prognostic markers. Whereas prognostic markers explain variability irrespective of treatment, our study intends to use predictive markers to explain outcome variability in response to treatment.

Gene expression profile using the microarray technology has led to a series of promising results through tissue gene expression profiling of different malignancies, including cancer. Interestingly, gene signatures have been used successfully as prognostic predictor for patients with colorectal carcinomas [20]. In this regard, Ghadimi et al. were able to predict response to therapy using gene expression profiles. Tumor behavior was correctly predicted in 83% of patients. Sensitivity (correct prediction of response) was 78%, and specificity (correct prediction of nonresponse) was 86% [21].

Similarly, it has been repeatedly demonstrated in recent years that genetic expression in BCs is altered in the context of malignancy. This observation of an altered BCs genetic expression profile in cancer patients was first reported in hematological malignancies. Today, current publications suggest that BCs could be valuable surrogate markers with diagnostic potential and prognostic applications in different cancer localizations such as renal, breast, esophageal, pancreatic and colorectal [12]–[16]. Nevertheless, to our knowledge, no publication has ever attempted to investigate the genetic profile of BCs as surrogate predictive markers of response to treatment in solid organs.

But which could be the rationale of our investigation? In this context, it has been proposed that tumor shrinkage is not simply dependent on direct damage to irradiated tumoral cells but that it is also greatly affected by the host immune response [22]. In fact, in vivo studies have suggested that cancer cells, dead or dying due to CRT, can present tumor-associated antigens to host immune cells and thereby evoke anti-tumor immune responses [23], [24]. Moreover, mounting clinical data suggest the presence of radiation-induced anti-tumor immunity in humans [25], [26].

Since lymphocytes, especially T cells, play a central role in anti-tumor immunity, Molling et al demonstrated that high levels of circulating invariant natural killer T (iNKT) cells predict the clinical outcomes of patients with head and neck squamous cell carcinoma [27]. Moreover, specifically for rectal cancer, Kitayama et al. [28] speculated, after observing that the percentage of lymphocytes showed a strong association with response to CRT, that the lymphocyte-mediated immune response against damaged tumor cells is critically important for achieving response.

Other investigations in rectal cancer patients, studying the increased apoptosis of lymphocytes in good responders to in vitro irradiation, suggest that the radiosensivity of malignant cells might be correlated with that of normal cells in rectal cancer and raise the possibility that the cancer response to CRT may be predicted by analyzing peripheral BCs [29].

The results reported here show that only a few genes among several thousand tested were differentially expressed with a statistically significant frequency between peripheral mononuclear cells of BCs from responder and non-responder LARC patients to CRT. Expression levels of CIR, PRDM2, CAPG, FALZ, NUPL2, and ZFP36 were higher in responder patients. The results from qRT-PCR showed trends that coincided with the microarray, although the only statistically significant changes in expression were for the FALZ gene (CIR and ZFP36 showing values close to significance level). The relationship between the FALZ expression and the effects of neoadjuvant treatment on rectal cancer has not been investigated. There are, however, evidences which suggest that they could be new molecular markers for predicting response to neoadjuvancy in rectal cancer patients. The FALZ locus codes various transcription factors, whose overexpression lead to apoptosis [30], and it is known that in many tumours, apoptosis is the main mechanism for the death of cancer cells in response to common treatment regimens. ZFP36 induce vascular endothelial factor (VEGF) mRNA degradation [31] and decreasing Ras-dependent VEGF expression [32]. The VEGF reduction has been related to prediction of efficacy of treatment with cetuximab plus weekly irinotecan in heavily pretreated advanced colorectal cancer patients, as well as overall survival [33]. However, further studies are needed to conclude whether FALZ, CIR and ZFP36 are involved in the response to therapy.

To investigate whether the gene expression profile in BCs is a representation of the genes expressed in the tissues themselves, we also looked for any similarity between the differentially expressed genes identified in our study and those observed by microarray analysis in tumor tissue of LARC patients (data not shown). We did not find any of the eight genes that were differentially expressed in BCs from responding and non-responding patients in gene expression patterns of LARC tissues (unpublished data). Thus it appears that, in general, gene expression in BCs does not mimic that in the primary tumor and, therefore, is not an artefact of circulating tumor cells.

On the other hand, while the addition of oxaliplatine to some patients could reflect a drawback of the study, it should be emphasized that the main treatment effect of RCT relies on radiotherapy. In fact, results from phase III studies [34] outline the importance of delivering 50.4 Gy independently of the chemotherapeutical agent employed (capecitabine alone or in combination with oxaliplatine).

## Conclusions

In the present study we analyses gene expression profiles obtained through whole genome-based microarrays in peripheral BCs samples from LARC patients to evaluate their utility as predictor of response to CRT. Our data show significant differences in gene expression profiling when comparing responders and non-responders. Using expression microarrays we have identified eight genes whose expression differed significantly between responders and non-responders. One of them, FALZ gene was corrobated using the qPCR, an independent assay and more accurate. Interestingly, FALZ has a relevant function in anti-tumour immunity.

As far as we know, the present study represents the first analysis of BCs gene profile from patients with LARC depending on response to CRT. Establishment of such differential expression has the potential to yield a rich compendium of potential genes for further pursuit as novel predictive markers. Moreover, the genes identified in our study could offer new insights into the immune system’s dysregulation in LARC.
